# Enhancing Health Innovation and Entrepreneurship Through an Entrepreneurial Fellowship Program

**DOI:** 10.1007/s10439-025-03743-w

**Published:** 2025-05-05

**Authors:** Carter Bloch, Mads Schäfer Bak, Sys Zoffmann Glud

**Affiliations:** 1 Danish Centre for Studies in Research and Research Policy (CFA), Department of Political Science, Aarhus University, Aarhus, Denmark; 2 BioMedical Design Programme, Department of Clinical Medicine, Aarhus University, Aarhus, Denmark

**Keywords:** Entrepreneurship education, Health innovation, Entrepreneurial mindset, Teamwork, Creativity

## Abstract

**Purpose:**

The purpose of this paper is to study how the BioMedical Design Programme affects and shapes individual development, group work dynamics among participating fellows and, through these, innovation processes and outcomes.

**Methods:**

Drawing on surveys of fellows in the period 2018–2023, it examines changes in fellows’ perceived levels of creativity and entrepreneurship and the role of team dynamics through surveys conducted before and after the fellowship.

**Results:**

The program led to perceived improvements in being creative, problem solving, collaborating, utilizing networks, and in taking risks. However, at the same time, the program left fellows with a deeper understanding of and respect for the challenges in creating a start-up. One year after the program, fellows reported benefits for their new employment or for their continued start-up ambitions, where around 60% were still involved in an entrepreneurial project.

**Conclusion:**

Our results confirm earlier work that shows that entrepreneurial education programs have a positive impact on entrepreneurial attitudes and intentions, where we are also able to explore in greater depth the ways in which the program impacted participating fellows.

## Introduction

The complexity of today’s healthcare requires a multidisciplinary approach to both solve the demands for new technology that can improve the sector’s ability to prevent, diagnose and treat patients, and to be able to meet the changes in demographics in the near future [24, 30].

It means that more specialists of the existing workforce, who traditionally have not thought of themselves as being part of healthcare innovation, need to acquire the competencies to help meet these challenges. These include creativity and entrepreneurial competencies along with the networking skills and the ability to work in multidisciplinary teams [27]. Innovative skills include the ability to identify unmet healthcare needs, ideation, development, and commercialization [29]. They also include the aptitude to navigate interdisciplinary environments across sectors and capacity to bridge academia, healthcare, and industry [1].

The BioMedical Design Novo Nordisk Foundation Fellowship Programme (BMD) was established in 2018, aiming to impact the future of healthcare through needs-driven innovation and product development by providing educational training for entrepreneurial talents. The ambition is to motivate the participants to create and realize innovative solutions to relevant, high impact, unmet needs in healthcare. The BMD is inspired by Stanford University’s BioDesign Fellowship and inscribes itself into a range of related programs across the world (TMC BioDesign; BioInnovate Ireland; Singapore BioDesign; Japan BioDesign; CfHE India). BMD trains fellows in clinical field observation, as well as creative and entrepreneurial skills. Alongside coursework, fellows work together in teams to develop and commercialize a project idea based on their observation work.

Beyond the program goal of developing entrepreneurs and innovations within healthcare, the program also seeks to contribute with new knowledge on how entrepreneurial skills, creativity, and innovative mindsets are developed and fostered. The focus on developing personal behavioral skills at BMD specifically addresses and increases individuals’ creative self-efficacy since this is a necessary precursor of creative efforts [3, 26]. Creative self-efficacy means bringing fellows to a level where they believe in their own abilities and resources to act creatively and produce creative outcomes in the medical device/health tech arena. A high level of self-efficacy is critical for motivation and actual capacity to engage in the pursuit of certain tasks and to have the perseverance to endure obstacles and long processes [3]. These are elements which are equally important for entrepreneurial activities—not to mention innovation itself [2].

While they can vary greatly in their design and structure, entrepreneurship education programs (EEPs) have a common goal in seeking to positively impact students’ entrepreneurial attitudes, intentions, and behaviors. In comparison with a control group, Gibcus et al. [14] find that participants in university entrepreneurship education programs had a greater entrepreneurial mindset, were more innovative in their jobs, and were more likely to start their own business. In addition, these programs enhance students’ knowledge about entrepreneurship, self-efficacy, and perceived behavioral control [4, 13, 19, 20, 22]. Team composition can also play a role for the impact of EEPs. Leatherbee and Katila [16] find that acceptance of EEP approaches depends on team composition, where team members with a business education background showed greater resistance to use of the lean start-up method in entrepreneurship training. Ceresia’s [8] literature review examines empirical evidence on how entrepreneurship education impacts entrepreneurial intentions, skills, and actual entrepreneurial outcomes. He concludes that the relationship between entrepreneurship education and entrepreneurial intentions is complex, with some studies reporting small positive effects.

Brinton et al. [6] outline the structure of the Stanford Biodesign Innovation Fellowship program, the outcomes in terms of the trainees’ career paths, and the technologies they have created. They find that fellows embark on a variety of new paths, many involving innovation or entrepreneurship in different ways. This includes leadership roles in companies that they founded directly from the program, start-up companies not directly founded within the program, involvement in medical technology innovation programs at other universities, and positions in large medtech companies. Wall et al. [28] also examine the career impacts of the Stanford program through a survey of previous fellows and outcome comparison with unsuccessful applicants to the program. They found that 60% of fellows were employed within health technology compared to 35% among unsuccessful applicants, and 67% of fellows felt that the fellowship had been ‘extremely beneficial’ on their careers. McGloughlin et al. [17] follow outcomes for the first fellows of the BioInnnovate Ireland program, finding that 10 out of 13 fellows were still involved in clinical innovation projects, and 4 out of 13 were still working on commercialization of their project initiated in the program.

The purpose of this paper is to study how the BMD program affects and shapes individual development, group work dynamics among fellows and, through these, innovation processes and outcomes. It examines changes in fellows’ perceived levels of creativity and entrepreneurship and the role of team dynamics through surveys conducted before and after the fellowship. In relation to other work on the role of EEPs, our analysis examines impacts on entrepreneurial intentions and attitudes and career and project outcomes using an approach that compares results before and after program participation. In addition, we examine the important role of team collaboration for these impacts.

The paper first provides a brief account of the BMD program followed by a methods section as well as a description of the data collection procedure. The results are thematically divided into the above-mentioned categories before a discussion and conclusion section rounds off the paper.

## Materials and Methods

A cornerstone of BMD is providing both education and hands-on experience with health innovation and commercialization to a diverse group of actors coming from healthcare, product design, engineering, business, and research. A noteworthy feature of the program is the expansion of the target group of participants beyond university students or researchers, to also include professionals from across the entire life science industry who function as an untapped resource of prospective innovators and entrepreneurs. They may have the ideas, but could lack the support, self-confidence, and/or daring to pursue a life as inventors and entrepreneurs in life science.

Through a 10-month program, the fellows are organized in multidisciplinary teams of 3–4 fellows in which they work together for the duration of the fellowship. The intermixture of academic backgrounds is done purposefully to train the fellows’ collaborative capacities and allow them to utilize the creative environment that can arise within a multidisciplinary group. From a pool of 100+ applicants, between 16 and 19 candidates are chosen each year for the program.

The program begins with a 4-week “boot camp” on needs-driven innovation processes, followed by 8 weeks of clinical immersion, where the fellows are provided access to a clinical department in order to observe and identify unmet needs for technological solutions. This phase allows the fellows to identify *actual needs* based on observations, rather than relying merely on interviews and expressed needs. The needs-based innovation approach builds partly on the widely acknowledged design thinking model for new product development (empathize, define, ideate, prototype, test) [7, 21].

During clinical immersion, hundreds of needs are typically identified, where only 2–3 are prioritized and brought to the next stage: the creative skills phase. In this phase, solution proposals for each of these needs are ideated, some ideas are prototyped, and in the end, each group selects one need with one product concept to go forward with to the commercial skills phase, where a business model and development and implementation strategy are drafted.

Commercialization skills include a range of strategic business elements, such as intellectual property rights, regulatory approval, health economics, business models, reimbursement, and so forth. They are necessary constituents to shape and foster entrepreneurs to launch new ventures with their eyes open to what comes ahead.

To build prior market knowledge and professional experience, the principles of get-out-of-the-building (adapted from Lean Start-up, [5]) are applied throughout the fellowship. The methodology is basically an iterative process of formulating very concrete assumptions, identifying people and stakeholders who may be able to qualify the truth of the assumptions and conducting semi-structured interviews to gather proof or disproof of the assumptions.

Data collection was based on a series of surveys among fellows that covered creative and entrepreneurial mindset, teamwork, and fellow and project status 1 year after graduation from the program. The surveys are conducted annually for each new group of fellows. The surveys on creative and entrepreneurial mindsets and on teamwork were both conducted during the program. These two surveys were conducted twice each year, at an early stage of the program (“pre”) and at the end of the program (“post”). Having responses at both the beginning and end of the program allows us to measure differences in perceived skills, confidence, and team effectiveness. An alumnus survey inquired about fellow and project status 1 year after the program. All survey questions are built on validated questionnaires from peer reviewed published articles.

The appendix lists the sources for each survey question used in this paper. Measures of entrepreneurial mindset are taken from Moberg et al. [18] and on creative self-efficacy from Tierney [25]. In both cases, a Likert scale is used, ranging from strongly disagree to strongly agree with each statement. Questions on managing ambiguity are based on Moberg et al. [18] and Chen et al. [9], where the former also uses a scale ranging from strongly disagree to strongly agree, while the latter uses a scale ranging from completely unsure to completely sure. Finally, questions on networking are from De Noble et al. [10] and Moberg et al. [18] and likewise use a scale ranging from strongly disagree to strongly agree.

**Fig. 1:**
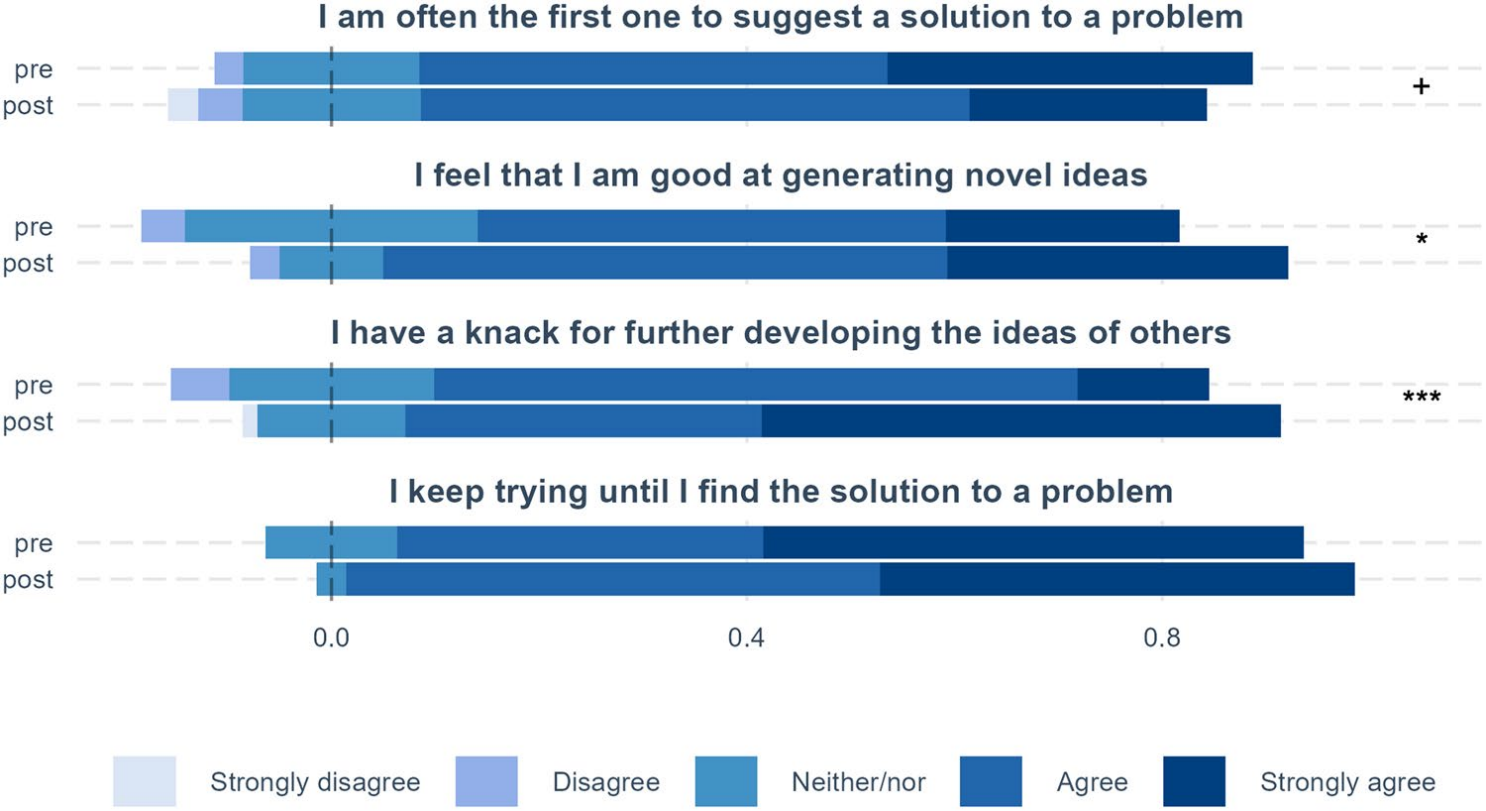
Creative self-efficacy. Distribution of responses at the beginning (pre) and end (post) of the program. The stippled line represents the median of the neither/nor and the scales show the share that is greater than the median. The significance of pre–post differences is shown on the right-hand side of the figure, where ***p < .001, **p < .01, *p < .05, ^+^*p* < .1.

**Fig. 2:**
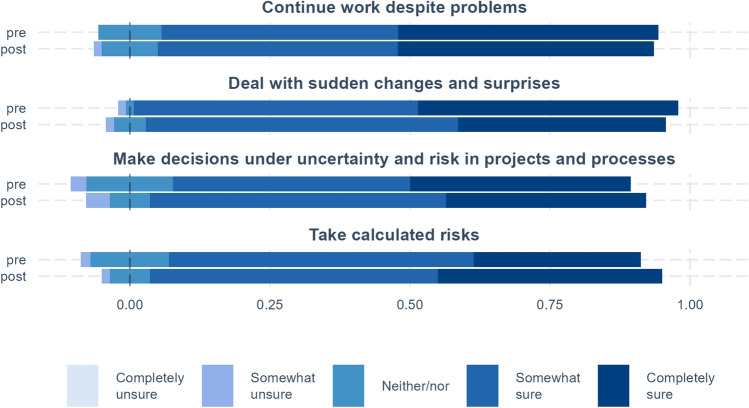
Managing ambiguity. Distribution of responses at the beginning (pre) and end (post) of the program. The stippled line represents the median of the neither/nor and the scales show the share that is greater than the median. The significance of pre–post differences is shown on the right-hand side of the figure, where ***p < .001, **p < .01, *p < .05, ^+^*p* < .1.

**Fig. 3:**
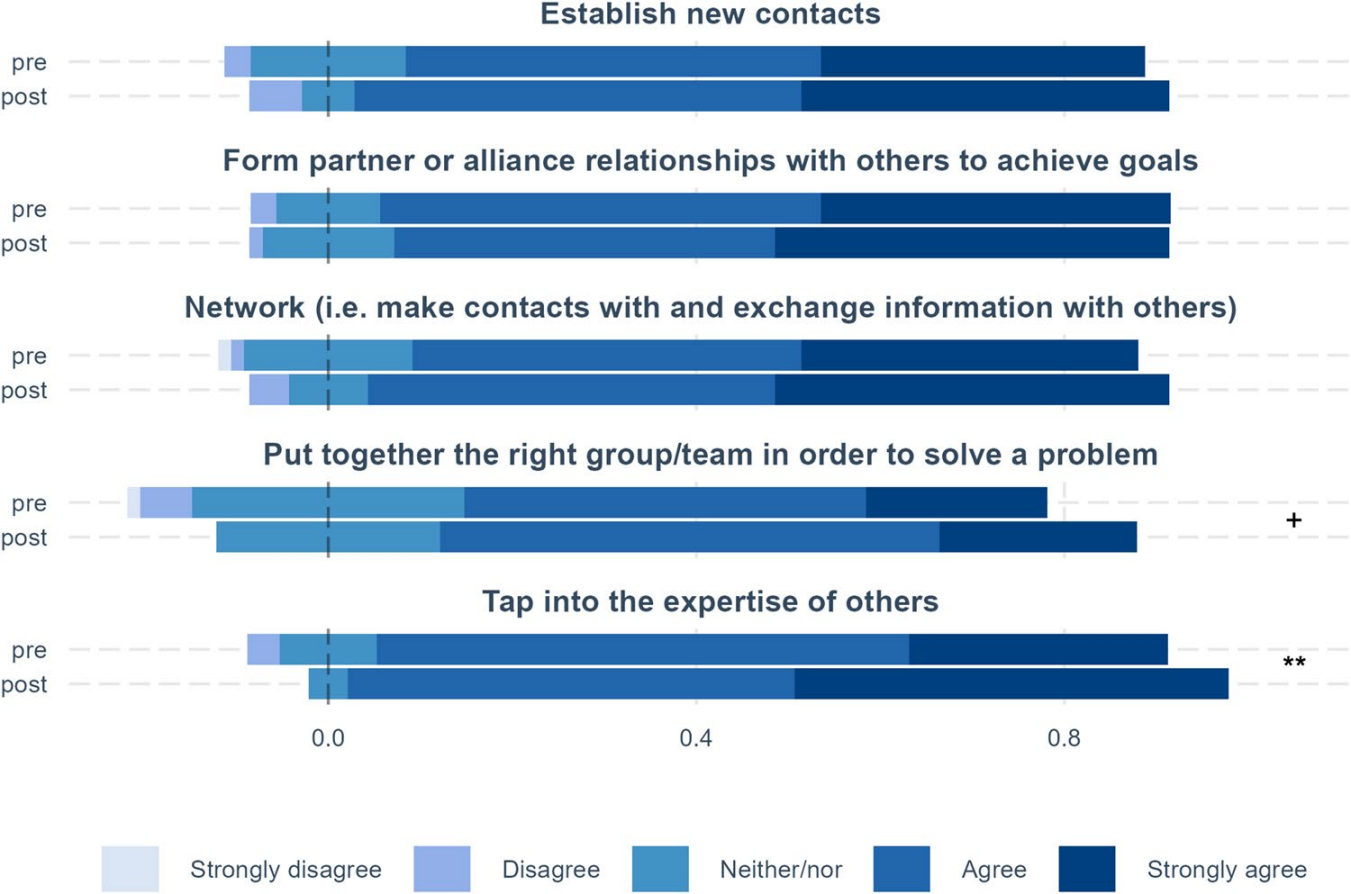
Networking. Distribution of responses at the beginning (pre) and end (post) of the program. The stippled line represents the median of the neither/nor and the scales show the share that is greater than the median. The significance of pre–post differences id shown on the right-hand side of the figure, where ***p < .001, **p < .01, *p < .05, ^+^*p* < .1.

**Fig. 4:**
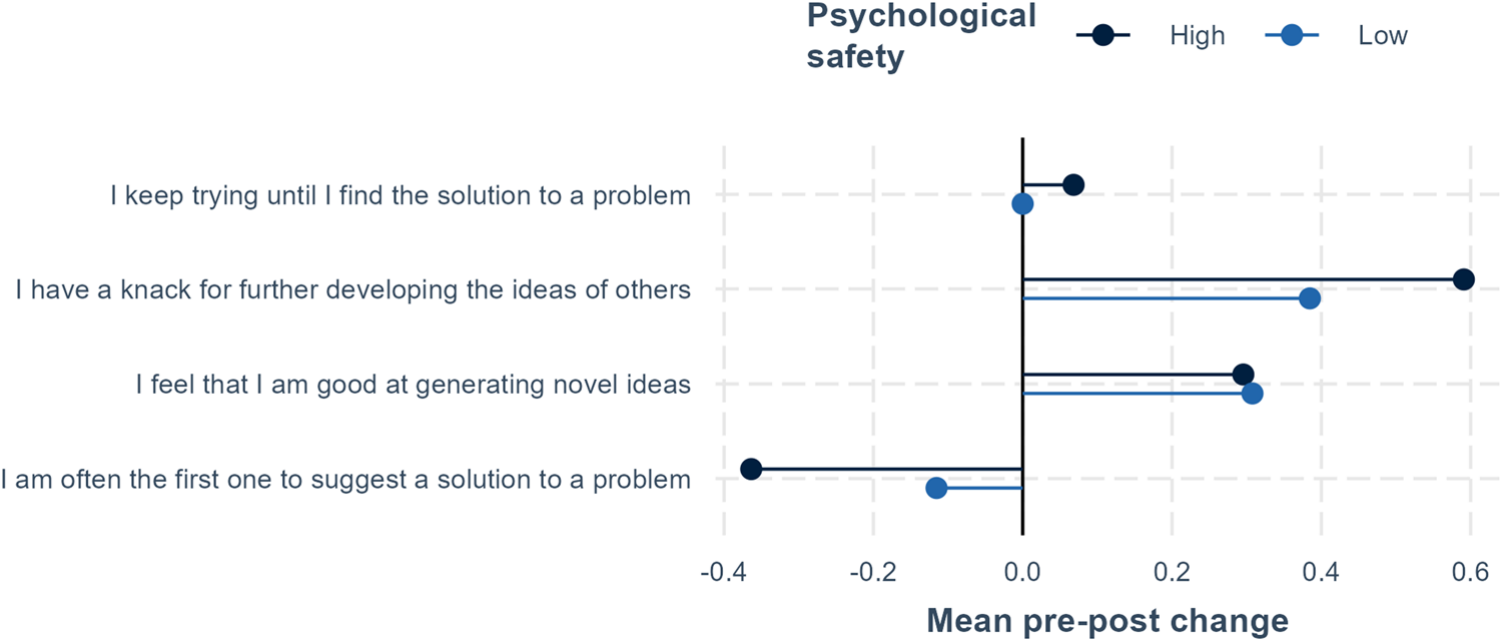
Creative self-efficacy and psychological safety. Average pre–post change for fellows in teams with high and low perceived psychological safety. The significance of pre–post differences is shown on the right-hand side of the figure, where ***p < .001, **p < .01, *p < .05, ^+^*p* < .1.

**Fig. 5:**
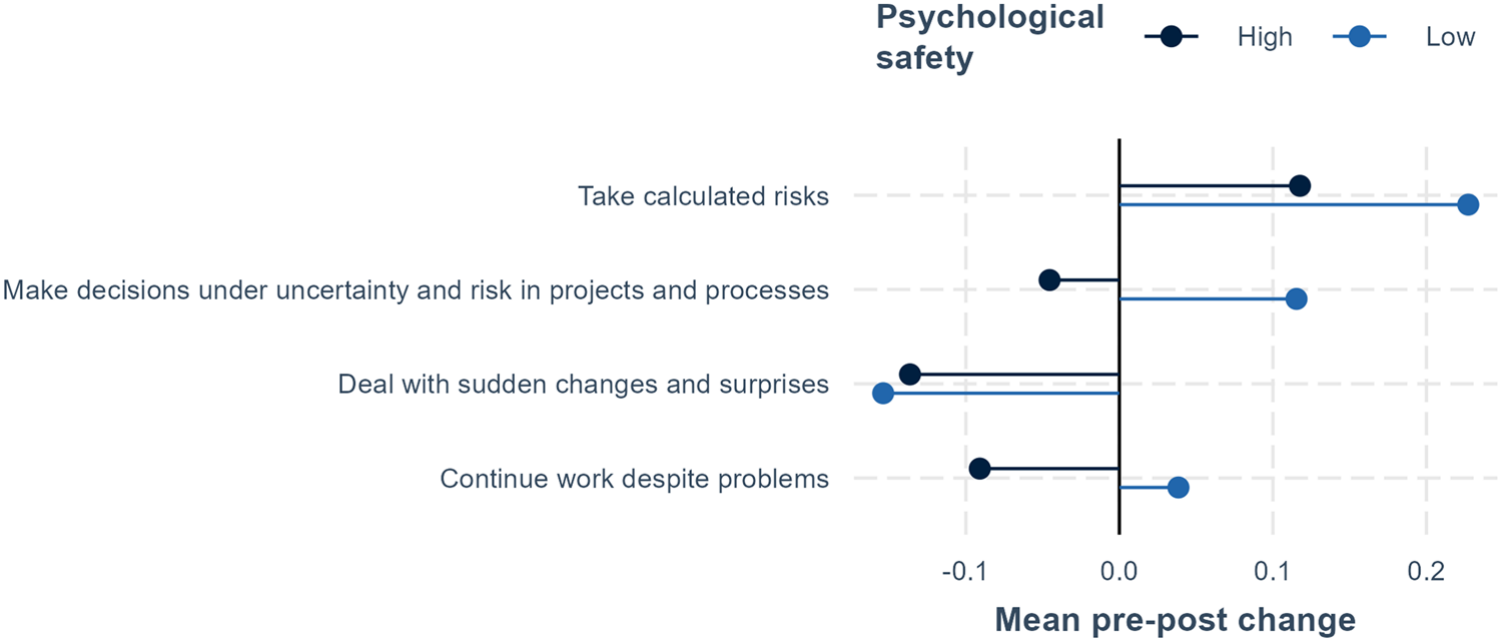
Managing ambiguity and psychological safety. Average pre–post change for fellows in teams with high and low perceived psychological safety. The significance of pre–post differences is shown on the right-hand side of the figure, where ***p < .001, **p < .01, *p < .05, ^+^*p* < .1.

For the topics entrepreneurial mindset, creative efficacy, managing ambiguity, and networking, we compare pre and post results (see Figs. 1, 2, 3). In addition, we calculate pre–post differences for each measure by quantifying the answer scales (e.g., strongly disagree equals 1 and strongly agree equals 5) and then subtracting the pre score from the post score. These measures are used to examine how these differences vary by the perceived psychological safety of fellows’ teams (see below and Figs. 4 and 5).

Measures taken from the alumnus survey, which was conducted 1 year after completion of the program, ask to what extent fellows think that they have obtained or improved their knowledge, skills, and competencies from the program within a range of areas, e.g., interdisciplinary collaboration and identifying market opportunities. The scale is based on four response options (to a large extent/to some extent/to a little extent/not at all).

### Psychological Safety

It is well-established knowledge that interdisciplinarity in teams can enrich and heighten innovative work [15]. However, fruitful teamwork and interdisciplinary collaboration are not a given result merely because of bringing competent people together in a project. While many elements are important when creating a successful team, this paper utilizes the concept of psychological safety to assess the role and impact of teamwork for individual development. Psychological safety is defined as the shared belief held by members of a team that the team is safe for interpersonal risk-taking, there is confidence in the team and mutual respect and trust among its members [11, 12].

Psychological safety is calculated based on the average of answers to seven questions taken from Edmondson [12] (see Appendix). All questions are based on a five-point Likert scale ranging from strongly disagree to strongly agree with each statement. In calculating the measure of psychological safety, each response is given a value (one for strongly disagree and five for strongly agree) and the average is taken across the seven responses for each fellow. Below, we compare individual skills and mindset development for experienced low and high psychological safety.

## Results

In the following, we first analyze survey results regarding the development of the fellows’ creative and entrepreneurial mindset as well as their ability to network. Second, the role and possible influence of teamwork for individual development are explored. Finally, we explore status and outcomes for individual fellows and their teams and projects.

### Fellow Statistics

There was a total of 71 fellows during the first 4 years of BMD. Almost all fellows responded to the survey during the fellowship (70 out of 71). Response rates for the alumnus survey are slightly lower, at 92%, and only cover the first three cohorts as the fourth cohort has just recently graduated.

Table 1 below shows some basic statistics for the sample. 41% of the fellows are women and around 45% have either a medical degree or a PhD. Almost half have a background in health or medicine with the remainder mainly within technical areas. Most fellows are Danish citizens, other nationalities making up 27% of the sample. Around 55% state at the start of the program that they often think about starting their business. The fact that this number is not 100% suggests that a significant share of fellows may join the program out of an interest in working with innovation but with less interest in becoming an entrepreneur.

**Table 1:** Basic characteristics of BMD Fellows

Fellow characteristics	% of fellows
Women	41%
MD	20%
PhD	25%
Masters	45%
Health	48%
Architecture and design	7%
Engineering and Tech	24%
Natural sciences	7%
Danish citizen	73%
EU citizen (not Danish)	14%
Non-EU citizen	13%
Often think about starting a business (agree or strongly agree)	55%

Given that the perceived effects of the program depend on the levels and abilities of fellows prior to the program, we show both the results at the start and completion of the program in order to gain an idea of the differences over time. Additionally, individual development may depend on how well teamwork functions, since fellows spend much of the program working on their entrepreneurial projects in teams. To explore this, we examine how pre–post differences for individual development vary according to the perceived psychological safety of their respective teams.

### Creativity and Entrepreneurial Mindset

A series of questions were asked in pre and post surveys to disclose developments in the creative and entrepreneurial mindsets of the fellows (Fig. 1), as well as their ability to manage ambiguity (Fig. 2). The surveys cover cohorts 1–4 (*n* = 70). In Figs. 1, 2, 3, results show the full distribution of responses to each listed question at the beginning (“pre”) and end (“post”) of the program. This allows us to see how perceived competencies and confidence have developed over the course of the program. The stippled line in the figures is set at the median of the neither/nor categories and the scales shows the share that is greater than the median. In doing so, the figures seek to show the full distribution of responses, while at the same time providing a visual comparison of shares of pre and post fellows that agree with statements. The figures also show the results of t-tests of whether there is a significant difference between average values for the beginning and end of the program.

For the first question, “I am often the first one to suggest a solution to a problem,” the proportion of respondents marking strongly agree diminished by over a third. More fellows also disagree with the statement, while some even strongly disagree which was not the case pre-BMD. This shift suggests that post-BMD, fellows are re-evaluating their own competencies, as well as their self-perceived role in relation to others and in a team, respecting other fellows’ competencies and being less inclined to believe they surpass others in capability. It may also imply an increased respect for the complexity of health challenges and an acknowledgment that a broad set of insights are needed before a resolution can be identified. It can be interpreted as a positive development, where fellows become more reflective, open-minded, and receptive to others’ suggestions and less inclined to believe they themselves know the best solution to a given problem. The difference is weakly significant at the 10% level.

The fellows’ perception of their own creativity skills has improved which is reflected in the significant shares that agree and strongly agree with statements concerning novelty and development, “I feel that I am good at generating novel ideas” and “I have a knack for further developing the ideas of others.” The latter, especially, displays a large improvement in the percentage of strongly agree. In both of these cases, the differences are significant. Adding to this is the (albeit prior high) positive though insignificant increase in perseverance toward problems contained in the question “I keep trying until I find a solution to a problem.” When combined, the answers suggest that fellows have obtained the necessary competencies to continue ideation processes. This improvement in creative self-efficacy is a vital part of the BMD because it is a key indicator of an enhanced innovative attitude and the ability to produce creative outcomes.

Looking at the fellows’ ability to manage ambiguity, Fig. 2 shows that most felt fairly confident pre-BMD, and the post-answers reveal little change. The high percentage of fellows answering “somewhat sure” or “completely sure” to these questions prior to the program suggests that the BMD participants have a high degree of self-confidence and a positive attitude toward handling problems and uncertainties already. This is not surprising per se, because the fellows are highly competent and selected from a pool of talented applicants.

It is, however, interesting that the fellows’ perceptions of their ability to manage ambiguity appear to decrease for “deal with sudden changes and surprises,” though noting that the pre–post difference is not significant. This could potentially be a result of having gained new insights into their own competencies and/or knowledge of the complexities involved in managing ambiguity on the scale of a BMD project which is at a whole new level. The experiences could have stimulated the fellows to reevaluate themselves during the program, making them less inclined to have that high self-assurance. As mentioned above, this can be a positive development, because fellows might approach ambiguous tasks and issues more judiciously.

### Networking

Figure 3 covers different perspectives of making and maintaining contacts and exchanging information. There is a noticeable improvement on several parameters. There are very slight, and insignificant, increases in “establish new contacts” and “form partner or alliance relationships with others to achieve goals.” The latter may suggest that fellows placed greater weight in their networking activities on reaching out to external contacts with specific expertise or market knowledge.

This also aligns well with the slightly larger increase in “network (i.e., make contacts with and exchange information with others),” which though is not significant. Concerning collaboration, fellows felt that they were better able to “put together the right group/team in order to solve a problem,” which is weakly significant. The largest increases are for “tap into the expertise of others,” both among those that agree and strongly agree with the statement. The pre–post difference is also significant. Hence, a number of fellows feel that they are now better able to utilize external contacts than prior to BMD, which also appears to be the largest takeaway in terms of networking abilities. These results are thus supportive to the overall approach of the BMD, where non-cognitive traits are just as important as cognitive, so that the fellows feel they have improved in their networking competencies.

### The Role of Teamwork for Individual Development

This section explores the relation between teamwork and individual development for program fellows. More specifically, we compare individual development for fellows that experience high compared to low psychological safety in their teams. Utilizing the measure of psychological safety described above, in Figs. 4 and 5, we compare mean pre–post changes in relation to low and high psychological safety. In contrast to the results shown in Figs. 1, 2, 3, in Figs. 4 and 5, we quantify responses for creative self-efficacy and managing ambiguity, where each response is given a value (one for strongly disagree and five for strongly agree). The figures then display the differences in average values from the beginning to end of the program. For example, a value of 0.4 indicates that the average post value was 0.4 higher than the average pre value.

Figure 4 shows mean pre–post differences in perceived creative self-efficacy for low and high psychological safety. For individual ability to generate novel ideas or perseverance in finding a solution, no difference is found between fellows that worked in teams with high vs. low perceived psychological safety, though average improvements in generating novel ideas are equal for the two groups. For relative comparisons with others, for example “I have a knack for further developing the ideas of others” or being “I am often the first one to suggest a solution to a problem,” there are slightly larger differences between high and low psychological safety, though these are not significant.

As mentioned above, there is a decline in individual perceptions of problem-solving ability compared to others, which could reflect increased recognition of the abilities of others. This increased recognition appears to be largest for fellows in teams with high psychological safety. Finally, increases in perceived ability to build on the ideas of others are highest among well-functioning teams with high psychological safety. It can also be noted that none of the differences shown in Fig. 4 are significant, though the number of observations per group in Figs. 4 and 5 is essentially halved since we divide the sample into low and high psychological safety (i.e., with 35 observations in each group, whereas the comparisons in Figs. 1, 2, 3 have 70 observations in each group, pre and post).

Figure 5 compares the results for dealing with uncertainty and psychological safety. Surprisingly, a greater share among those in teams with low psychological safety felt they improved their abilities to “take calculated risks” and to “make decisions under uncertainty and risk in projects and processes.” One possible explanation for this result is that fellows in teams with low psychological safety may on average face more challenging uncertainties and risks to which they must act. Both groups felt a decline in their ability to “deal with sudden changes and surprises.” Those with low psychological safety appear to have a slight improvement in ability to “continue work despite problems,” while there is a decline for fellows with high psychological safety. This could reflect that in teams who were more challenged in establishing teamwork and making them function, some fellows felt that this strengthened their skills to work under less-than optimal conditions. Also here it should be noted that differences are not significant.

**Fig. 6:**
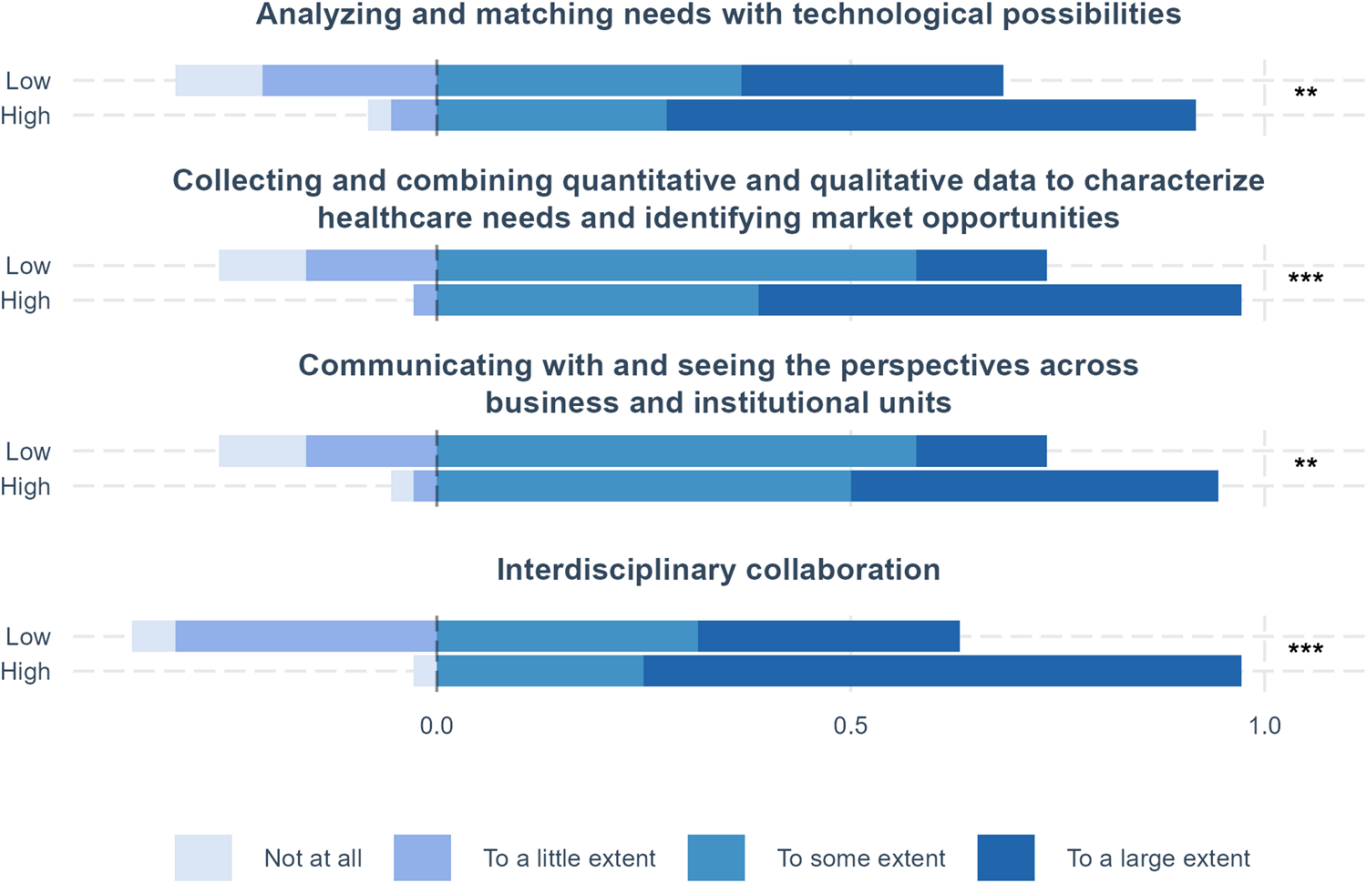
Perceived improvements in competencies 1 year after the BMD program. The significance of pre–post differences is shown on the right-hand side of the figure, where ***p < .001, **p < .01, *p < .05, ^+^*p* < .1.

Fellows were also asked to assess their improvement in skills and competencies 1 year after program completion. Figure 6 below shows the distribution of responses concerning different skills and competencies, showing whether fellows have experienced an improvement, from ‘to a large extent’ to ‘not at all.’ As above, we compare results for high and low psychological safety. In all cases, the differences between average results for high and low psychological safety are significant. First, we can note that overall, a far majority of fellows reported improvements to a large extent or to some extent. While this is the case for both high and low psychological safety, there are marked differences according to psychological safety for all four competencies. Between 90 and 100% of fellows in teams with high psychological safety reported improvements in competencies to some or a large extent, compared to 60 to 75% for fellows from teams with low psychological safety. Differences are even larger when only focusing on improvements to a large extent.

### Employment and Project Status 1 Year After the Program

This section draws on an alumnus survey 1 year after the program to examine employment and project status. The alumnus survey was answered by 48 alumni.

When asked about their current employment status, 25% (*n* = 12) answered they were employed in their previous job; 54% (*n* = 26) were employed in a new job; 23% (*n* = 11) were self-employed (full or part-time); while 10% (*n* = 5) were unemployed, on leave, etc. Note that a few alumni were both self-employed and working in an old/new job. It is salient that more than half of the alumni are employed in a new job at this point. Although other factors can play into this, it indicates that their participation in BMD has enhanced their competencies and their overall attractiveness in the pool of employers. The share of self-employed is also relatively high, suggesting that alumni have gained the skills, motivation, and courage to continue with their own entrepreneurial journeys.

Turning the attention to project status, 58% of participants (*n* = 28) were still involved in (active) projects when answering the survey. In terms of funding, 43% (*n* = 12) had secured some form of funding after 1 year, 25% (*n* = 7) were applying, and the remaining 32% (*n* = 9) had not applied for funding.

While a fairly high number are still involved in active projects, the evenly distributed numbers in terms of funding, or lack of, highlight the difficulty in gaining ground with a new project. Indeed, this is also reflected in the survey, where the alumni provide very diverse descriptions of their projects’ status, ranging from very early state prototype development to near complete product.

Around 42% of responding participants (*n* = 20) were not active in their BMD projects 1 year after the program. A variety of different reasons were given for why they had stopped, though most could be placed in one of three categories: the project was not considered to have sufficient potential, difficult team dynamics, and personal reasons not related to their team or project.

Although the projects are at different stages of development, more than half of the alumni (*n* = 16), who are still involved in projects, describe their career ambitions as “to go full time/make a living of it.” Recurring themes for these 16 alumni are a pronounced risk willingness (e.g., to put their own money into the project; to quit their current job or turn down other job offers) and a clear intention to go full time with their entrepreneurial projects when sufficient funding is in place. This entrepreneurial mindset is further illustrated by those who did not contemplate their career ambitions in terms of work status but rather emphasized their ambitions as entrenched in the patients and product development, e.g., one alumnus explains that she “really want[s] to see the project come to the benefit of patients. For myself, I see it as a steppingstone to a start-up,” while another describes his ambitions as “[solving] tangible problems and produce products that people actually use and make a positive impact in their lives.”

These are reoccurring answers which add to the overall picture wherein the majority of alumni have high entrepreneurial ambitions 1 year after the BMD program has ended. This applies to their personal attitude and approach to entrepreneurship, but also in terms of product development and innovation, where they display a creative mindset and a firm belief in having something to offer.

The survey also allowed for open responses to several questions, including a question on how participation in the BMD fellowship has influenced their tasks in their present job. Roughly three out of four noted that participation in the BMD had a positive influence on their current job tasks to a greater or lesser extent. Obviously, this is a favorable aspect, but what is more interesting is how varied the responses of the alumni are. While many of the answers were positive in the general sense, e.g., “I have some basics to build upon,” others answered more task specific. One notes how “I am the only one in my company that talks directly to the end-users, and by now it has proven its value as we have gained a lot of new insights about their needs.” Another emphasizes how she “benefits a lot from [her] improved confidence in managing innovation processes,” while a third described how she has become “more proactive in idea thinking.”

These comments on task specific improvements suggest that BMD participants have benefitted individually from the program on a rather broad spectrum, ranging from personal capacities to consumer and stakeholder interactions.

## Discussion

The purpose of the BioMedical Design Programme is to train and motivate participating fellows to be entrepreneurs and innovators. This paper has sought to investigate the short-term results of the program after its first 4 years, examining the impact of fellows’ skills and competencies. Given that the selected fellows are very talented prior to entering the program, we use a before–after approach to examine changes in perceived ability. In addition, we have also followed fellows 1 year after program completion. Our results confirm earlier work that shows that EEPs have a positive impact on entrepreneurial attitudes and intentions, where we are also able to explore in greater depth the ways in which the program impacted participating fellows.

Our survey-based analysis found improvements in creative self-efficacy, in ability to manage uncertainty and change, and in maintaining and utilizing network connections. In other cases, we found a decline in confidence in their ability to solve any problem or in performance relative to others. Our interpretation of this is that, through the challenges that fellows faced, they gained a greater understanding of what it really takes to develop an idea and to start a business, as well as greater recognition of the abilities of others and their need for support and collaboration. Some fellows may have found the context of innovating for the health sector with all its complexity and constraints to be too challenging for their personal preferences.

Teamwork was an important element of the program, with teams working intensively to develop and commercialize innovative health solutions. Each fellow gained valuable experience with working closely in a team and the challenges in establishing effective collaboration. Our results also show that how well a team functioned in terms of psychological safety influenced perceived individual skills gains.

The program also appears to have led fellows to reassess their abilities in some cases. The slightly lowered belief in “being the first to suggest solution to problem” can be explained as an outcome of discovering the value of getting more insights on a given situation before suggesting solutions, reflecting a development of humbleness to the complexity of entrepreneurship. Likely, it is also a learning outcome of being part of an extended and designed innovation process, where fellows will have witnessed multiple times that the first idea does not always prove to be the best.

The increase in belief in own abilities to generate novel ideas could be the result of having learnt to use specific tools and to follow structured processes for generating ideas. Also, since the fellows have experienced how they perform in a group during co-creation they have seen how their own contribution is to idea generation compared to others. These experiences of co-creation also probably contribute to an enhanced perception of being able to further develop the ideas of others. Perhaps they learn that it is not a competition to be first with the best idea, but that ideas need to undergo evolution to become viable and novel. Somewhere along that process they contribute to the improvement of the idea.

The minor shift in “ability to deal with sudden changes and surprises” toward less self-assuredness could be an effect of the character of market validation activities that are taught and trained in the program. Such activities (playing a role to de-risk the projects) lead to insights that almost on a bi-weekly basis rescope the project or lead to major pivots. In some cases, it even leads to scrapping a project completely and the team will have to start all over again. Presumably, a few fellows have experienced these shifts to a level where it has become uncomfortable for them to deal with.

It is a genuine entrepreneurial trait to be able to manage uncertainty and risks. It is reasonable to think that the fellows overall display a high level of competencies in managing ambiguity since the selection process for the program is geared toward ability to manage uncertainty and it demonstrates some calculated risk willingness to put one’s career trajectory on hold in order to participate in the program. This is particularly relevant for the first two cohorts where there was at that point no data available on what opportunities the program could provide them.

A lemonade principle [23] is promoted through the program’s “agility and get out of the building principle,” where fellows must test their assumptions out in the real world with real users or customers or stakeholders—this often leads to unexpected information which opens up new, but unexpected avenues for the project. These experiences also likely strengthen the perception of what uncertainties are (no historical data to lean on for decision making) and how to deal with them (harvest live insights and you can decide and design the future).

Another valuable outcome that arises from the practical ‘get out of the building’ activities in the program is the ability to establish new network contacts, exchange information with them, and subsequently apply the expert knowledge received to the project. It is reasonable to think that the increased self-belief in networking ability also lifts the belief in ability to put the right group of people together to solve a problem as seen in Fig. 3.

The questions in the alumnus survey 1 year after the program aimed to address the many learning objectives in the program in a condensed form to assess to which extent the learnings persisted after the program and thus could be viewed as robust. These learnings seem to be mirrored by the behavior and the roles fellows take on post-program.

One year after the program, almost all fellows felt that they had gained from the program and were putting their learnings to use in different ways, some as entrepreneurs, others as intrapreneurs that engage in entrepreneurial or innovation activities within a firm or public organization, and some as both. The majority were still involved in their project, some full time and others in their free time. This demonstrates sustained learning outcomes from which the individuals can develop further and create impact on health innovations and ecosystems through continuous application of the acquired skill set.

In discussing the survey results, it is also important to keep in mind that our sample size is relatively small. When comparing before and after results, as in Figs. 1, 2, 3, we have in all 140 observations for 70 persons. However, for the comparisons of low and high psychological safety in Figs. 4, 5, 6, total observations are halved to 70, one observation per person. Small sample sizes can lead to greater uncertainty in estimates, potentially affecting the statistical precision of some results.

## Conclusion

The impact of lower psychological safety in teams on individual learnings is being addressed in the future development of the program through increased focus on preventive team sparring sessions with professional occupational therapists and increased awareness from the instructors on facilitating team development conversations using different means such as team canvas models.

The pre–post surveys on creativity and entrepreneurial mindset as well as the 1-year follow-up will be continued throughout the next 5 years. A significant change to the program was made through the introduction of strategic innovation challenges set by the collaborating hospitals. Whereas the teams in the first 4 years of the program (which is the basis for the results presented in this paper) had freedom to choose unmet needs, the future fellow teams will only be able to select unmet needs that comply with the strategic scope of the innovation challenge. It shall be interesting to see how these changes influence the outcome of creative self-efficacy and entrepreneurial mindset. Further research developments will aim to assess the quality of the innovation created by the fellow teams by evaluating the innovativeness of the final projects and then compare how quality in projects correlates to team psychological safety and individual learning outcomes. In addition, the study plans to follow fellows and projects over a longer period to time, in order to better understand how the program contributes to their longer-term success.

## Appendix

**Table:**

Survey questions	Source
Creativity and entrepreneurial mindset/self-efficacy survey questions	
“I have confidence in my ability to solve problems creatively”	Tierney [25]
“I have a knack for further developing the ideas of others”	Tierney [25]
“I feel that I am good at generating novel ideas”	Tierney [25]
“I am often the first one to suggest a solution to a problem”	Moberg et al. [18]
“I keep trying until I find the solution to a problem”	Moberg et al. [18]
“I believe failures allow opportunities for reflection and consideration”	Inspired by Moberg et al. [18]
Managing ambiguity survey questions	
“Work under pressure and conflict”	Chen et al. [9]
“Take calculated risks”	Chen et al. [9]
“Make decisions under uncertainty and risk in projects and processes”	Chen et al. [9]
“Deal with sudden changes and surprises”	Moberg et al. [18]
“Continue work despite problems”	Moberg et al. [18]
Networking: “I am able to…”	
“Develop and maintain favorable relationships with potential investors”	De Noble et al. [10]
“Establish new contacts”	Moberg et al. [18]
“Form partner or alliance relationships with others to achieve goals”	Moberg et al. [18]
“Network (i.e., make contacts with and exchange information with others)”	Moberg et al. [18]
“Put together the right group/team in order to solve a problem”	Inspired by De Noble et al. [10]
“Tap into the expertise of others”	Inspired by De Noble et al. [10]
Improvement in skills and competencies (1 year after program completion) questions	
“Interdisciplinary collaboration”	Based on BioMedical Design’s own learning goals. https://biomedicaldesign.dk/learning-outcomes/
“Communicating with and seeing the perspectives across business and institutional units”	Based on BioMedical Design’s own learning goals. https://biomedicaldesign.dk/learning-outcomes/
“Collecting and combining quantitative and qualitative data to characterize healthcare needs and identifying market opportunities”	Based on BioMedical Design’s own learning goals. https://biomedicaldesign.dk/learning-outcomes/
“Analyzing and matching needs with technological possibilities”	Based on BioMedical Design’s own learning goals. https://biomedicaldesign.dk/learning-outcomes/
Psychological safety	
“If you make a mistake in this team, it is often held against you”	Edmondson [12]
“Members of this team are able to bring up problems and tough issues”	Edmondson [12]
“People in this team sometimes reject others for being different”	Edmondson [12]
“It is safe to take a risk in this team”	Edmondson [12]
“It is difficult to ask other members of this team for help”	Edmondson [12]
“No one in this team would deliberately act in a way that would undermine my efforts”	Edmondson [12]
“Working with members of this team, my unique skills and talents are valued and utilized”	Edmondson [12]
